# Experimental Study on Damage Identification of Nano-SiO_2_ Concrete Filled GFRP Tube Column Using Piezoceramic Transducers

**DOI:** 10.3390/s20102883

**Published:** 2020-05-19

**Authors:** Xixiang Chen, Yu Chen

**Affiliations:** 1 College of Technology & Engineering, Yangtze University, Jingzhou 434020, China; chenxixiang1979@163.com; 2 College of Civil Engineering, Fuzhou University, Fuzhou 350116, China

**Keywords:** glass fiber reinforced polymer (GFRP) tube, Nano-SiO_2_ concrete, piezoceramic tranducer, energy, damage

## Abstract

This paper proposes a new approach to damage detection of nano-SiO_2_ concrete-filled glass fiber reinforced polymer (GFRP) tube column using piezoceramic transducers. Stress waves are emitted and received by a pair of piezoceramic transducers embedded in the concrete-filled GFRP tube, and the energy and damage indices at different levels of loading in the tube are obtained by wavelet packet to evaluate the damage degree of GFRP tube nano-SiO_2_ concrete column. Through the experimental studies, the effects of different nano-SiO_2_ contents, concrete grades, and superplasticizer on the damage were analyzed to gain load–displacement curves, load–energy index curves, and load–damage index curves. The results show that the wave method can be adopted to monitor the damage of GFRP tube nano-SiO_2_ concrete column. The specimens with 3% nano-SiO_2_ content have the smallest energy change rate, indicating that adding 3% nano-SiO_2_ content into concrete can effectively delay the development of damage. After the addition of superplasticizer, with the increase in the strength grade of concrete, the cracks in the specimen tend to develop slowly, and therefore the specimens have a stronger resistance to damage. The damage of the specimens with the nano-SiO_2_ content of 1% appeared the latest, while the damage without the nano-SiO_2_ specimen appeared the fastest. The experimental results show that this method can better monitor the damage of the Nano-SiO_2_ concrete in the glass fiber reinforced polymer (GFRP) tube.

## 1. Introduction

Nano-SiO_2_ has been widely used in optics, electronics, biomedicine, building materials and photonic crystals because of its unique physical, chemical and structural characteristics due to its small size [1,2,3]. The fabrication, characterization, and application of cobalt ferrite (CoFe_2_O_4_ MNPs) nanocatalysts (CF-MNPs) in organic reactions were presented by Kaz et al. [4]. The studies on nanotechnology in drilling, reservoir protection, sensing and imaging techniques in oil and gas migration and accumulation were summarized by Zhang et al. [5]. The preparation method of CuO-ZnO nanocomposite was mentioned by Das et al. [6]. Antibacterial potential of nanocomposite-based materials was proposed by Kar et al. [7]. The characteristics and preparation of titanium dioxide nano photocatalysts were presented by Ramos-Delgado et al. [8]. At present, the research on nano-SiO_2_ concrete has become more and more extensive. The mechanical properties and durability of concrete can be improved by adding appropriate amounts of nano-SiO_2_ [9,10,11].

Traditionally, concrete is often reinforced with steel rebars or integrated with other steel components, such as tubes [12,13,14] and plates [15,16]. Since steel components are subject to corrosion [17,18,19], fiber reinforced polymer (FRP) materials, which have strong corrosion resistance [20,21], are increasingly used with concrete structures in different forms [22,23,24]. Among them, a glass fiber reinforced polymer (GFRP) tube concrete column is a composite structure formed by pouring concrete into GFRP tube. Compared with other restrained concrete members, a GFRP tube concrete column has many advantages such as strong bearing capacity, good integrity, high ductility, good corrosion resistance, and easy construction [25], thus having been widely applied to pile foundation structure, load-bearing column structure and bridge pier structure in civil engineering. However, due to the factors of environment and service life, cracks in concrete can seriously affect the safety of the structure. Hence, it is of immense significance to monitor the health of concrete columns. In recent years, piezoceramic materials have been actively used in structural health monitoring (SHM) owing to their high sensitivity [26,27,28], fast response [29], dual functions of sensing and actuating [30], and energy harvesting [31], and piezoceramic transducers have the capacity to generate and sense stress waves. Since stress waves, including ultrasonic waves, are important tools in SHM such as active sensing methods [32,33,34], piezoceramics-based SHM and damage detection are receiving increasing attention [35,36,37].

Currently, many scholars have conducted extensive research on the health monitoring of civil structures using lead zirconate titanate (PZT) transducers [38,39,40,41]. PZT is a type of piezoceramic material with strong piezoelectric effect. Kar et al. [42] conducted an experimental study for the damage assessment of concrete reinforcing bars using piezoelectric sensors. Song et al. [43,44] developed novel PZT-based multi-functional smart aggregates, which can be embedded in concrete structures. Furthermore, Song et al. conducted exploratory work on SHM of concrete structures using the smart aggregate technology [45,46]. Feng et al. conducted research on using embedded PZT transducers for concrete early age hydration monitoring [47]. In addition, Feng et al. applied smart aggregates to pile damage detection [48]. Jiang et al. investigated debonding between a FRP bar and concrete structures using PZT transducers [49]. PZT transducers are also used to monitor the grouting quality of the tendon duct of prestressed concrete beams [50]. Kong and Song compared the performances of shear and compressive smart aggregates [51]. Kong et al. monitored the cyclic cracks of a concrete structure using embedded PZT transducers [52]. Ma et al. [53] contrasted the measured signal amplitude of the piezoelectric sensor under harmonic excitation and the wavelet packet energy under frequency-sweep excitation at various grades of load, defined corresponding damage indices, and realized the effective monitoring of the damage status of bamboo beam members during loading. Xu et al. [54] fabricated embedded piezoelectric functional elements from piezoceramic plates and pre-embedded them in the specimens of concrete-filled steel tubular column with local interface damage, so as to monitor the bonding quality of the interface between the concrete and the steel tube wall. Meng et al. [55] proposed a statistical algorithm for the damage identification of concrete structures using piezoelectric smart sensors, in order to fulfill dynamic monitoring and damage evaluation in the specimen damage and destruction process. Yi et al. [56] adopted ANSYS simulation to study the damage identification of reinforced concrete structures based on PZT-guided wave method.

Even so, few scholars have studied the damage identification of GFRP tube nano-SiO_2_ concrete columns. When the GFRP tube nano-SiO_2_ concrete column is in the natural environment, the concrete in the GFRP tube will inevitably produce tiny cracks under the influence of loads and various emergencies (earthquake, typhoon, etc). As time goes on, the continuous increases in such cracks will greatly reduce the strength and stiffness of the structure, causing great harm to the whole structure. Hence, this paper intended to systematically study the damage identification of GFRP tube nano-SiO_2_ concrete columns by the wave method, perform wavelet packet analysis to calculate the stress wave energy before and after concrete column damage, and then gain the relative damage index to reveal the damage of the concrete in GFRP tube. Moreover, the influences of concrete grade and nano-SiO_2_ content on the damage of GFRP tube nano-SiO_2_ concrete column were analyzed, and the appropriate content of nano-SiO_2_ in concrete was suggested, which has not only profound theoretical importance but also significant engineering application value for the application of piezoceramics in the field of structural health monitoring.

## 2. Experiment Principle

### 2.1. Piezoelectric Effect of Piezoelectric Materials

The piezoceramic material refers to a crystalline material that generates charge transfer on the surfaces of both ends when subjected to pressure, as well as being a kind of smart material with the characteristics of self-perception, self-diagnosis, and self-repair. All piezoelectric materials have a positive piezoelectric effect and a negative piezoelectric effect. The positive piezoelectric effect refers to the fact that when a piezoelectric material deforms because of mechanical force applied, it can cause the internal positive and negative charge centers to move relative each other and cause polarization. As a result, charges with opposite signs appear on both surfaces of the material, and the charge density is proportional to the external force. The polarity of the charge changes with the direction of the external force, converting mechanical energy into electrical energy. Piezoceramic materials can be made into sensors using the positive piezoelectric effect, which have the advantages of fast response speed, wide frequency response range, and simple fabrication. The sensors can be used in health monitoring systems to detect structural damage sensitively and determine the location and size of the damage [57,58]. In contrast, the negative piezoelectric effect refers to the fact that the action of the electric field makes the materials deform and converts electrical energy into mechanical energy. A piezoelectric material can be made into a driver using the negative piezoelectric effect, which can be embedded in the structure or pasted on the surface of the structure as well as deform the structure or change the stress state of the structure [59].

### 2.2. Piezoceramic-Based Stress Wave Method

**Figure 1:**
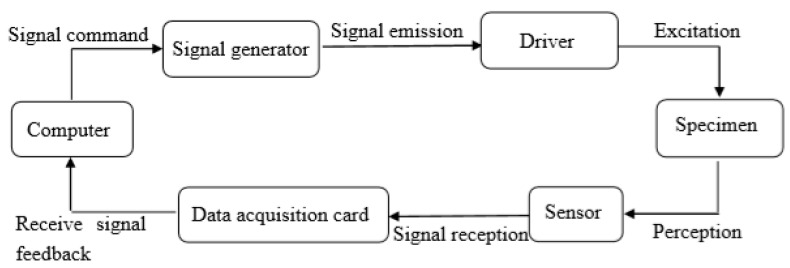
Block diagram of health monitoring system based on wave method.

In the use of piezoelectric materials for structural health monitoring and damage identification technology [60,61], there are mainly two methods of active health monitoring and passive health monitoring. The active monitoring method based on piezoelectric materials primarily focuses on impedance method and wave method [62]. The impedance method generally attaches the piezoceramic plate to the structural surface, which is easily affected by environmental factors, making the monitoring inaccurate and the internal damage of the structure difficult to monitor. Conversely, the wave method has the advantages of large monitoring interval, abundant parameters for damage analysis, and strong resistance to disturbance, and therefore it has been widely applied in the field of health monitoring. The basic principle of the wave method is to fabricate a pair of piezoceramic sensors into smart aggregate and embed them inside the structure. One sensor acts as a driver and emits stress waves which are transmitted inside the structure under test. Another piezoelectric ceramic sensor at a certain distance receives the stress waves and converts the measured stress waves into electrical signals for output. Figure 1 shows the basic composition of a health monitoring system based on the wave method. By comparing the parameters of the signal before and after damage, including the amplitude, energy, phase, and time delay of the signal, the health of the structure can be judged, and the damage location and degree of the structure can be confirmed [63]. On the basis of the principle of stress wave propagation in concrete, the stress wave energy decreases with the increase in the damage, so that the real-time damage monitoring of the structure can be carried out through the change trend of the stress wave energy during the process of damage development.

### 2.3. Wavelet Packet-Based Damage Identification Approach

Traditional signal analysis and processing methods generally use Fourier analysis, which is a fixed window function analysis method. It plays an irreplaceable role in the processing of stationary random signals. However, a large number of real signals are non-stationary time-varying signals. Traditional Fourier analysis cannot reflect the characteristics of a signal, such as non-stationary, short duration, time-domain and frequency-domain localization. The traditional Fourier transform processing is not ideal for non-stationary signals and hence it has been improved, which promotes the development of wavelet theory analysis. The developed wavelet theory analysis can perform multi-resolution analysis and solve many difficult problems in Fourier analysis, so it is an excellent tool for time domain analysis and frequency analysis of signals. Wavelet analysis is a time-frequency localization analysis method which can change time and frequency window. As it only decomposes the low-frequency signal in the process of decomposition, and does not decompose the high-frequency signal, its frequency resolution decreases with the increase in frequency [64]. The wavelet packet theory analysis method developed on the basis of wavelet theory analysis makes up for the characteristic of the wavelet theory analysis that the high-frequency signal cannot be deeply decomposed, and realizes the complete analysis of the signal [65,66]. The wavelet packet theory analysis has been widely applied in the field of time-frequency signal analysis and processing, such as single-dimensional signal recognition, signal noise elimination, and feature extraction. Due to the existence of damage in the structure, the amplitude of the received signal is greatly attenuated, and the energy of the signal is a physical quantity proportional to the quadratic power of the amplitude of the signal, so the health status of the structure can be identified by the change of signal energy. In this experiment, damage indices based on the wavelet packet analysis method were used to evaluate the damage severity of the GFRP tube concrete column. 

Firstly, the received original signal (Si) of the ith measurement was decomposed into 2^n^ signal subsets with different frequency bands. The signal subset is X_i,j_, where j is the frequency band (j = 1, 2, …, 2^n^), which can be expressed as:

X_i,j_ = { X_i,j,1_, . . . , X_i,j,m_} (1)

In the above formula, m is the number of data samples that decompose the signal subset.

Secondly, the energy of the jth sub signal in each band of the N-layer signal can be defined as:

(2) $$e_{\mathrm{ij}}={X^{2}}_{i,j,1}\;+{X^{2}}_{i,j,2}\;+\;.\;.\;.\;{X^{2}}_{i,j,m}$$

The total energy of the original signal Si after wavelet packet decomposition and reconstruction is defined as:

(3) $$E_{\mathrm{ij}}=\sum_{j=1}^{2^{n}}e_{\mathrm{ij}}$$

In structural health monitoring, the damage degree of structure or component is defined as damage index, which is a quantitative measure. Generally, the parameters related to the structural characteristic parameters, such as the elastic modulus and the energy of monitoring signal, are selected to judge the damage degree quantitatively [67]. Finally, the damage index can be defined as [68]:

(4) $$H=\sqrt{\frac{\sum_{j=1}^{2^{n}}{\left(E_{0,j}-E_{i,j}\right)}^{2}}{\sum_{j=1}^{2^{n}}E_{0,j}^{2}}}$$

where $\overset{2^{n}}{\underset{j=1}{\sum }}E_{0j}$ stands for the energy in the healthy state, and $\overset{2^{n}}{\underset{j=1}{\sum }}E_{\mathrm{ij}}$ stands for the energy the in damaged state. The higher the damage index H is, the higher the damage degree of the specimen. When H = 0, it means that the specimen is not damaged, but when H = 1, it means that the specimen is completely destroyed.

## 3. Experiment Scheme

### 3.1. Specimen Design

**Figure 2:**
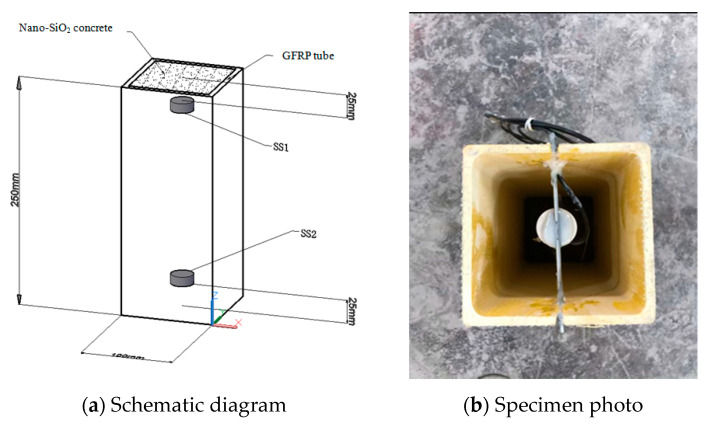
Schematic diagram and specimen photo of short glass fiber reinforced polymer (GFRP) concrete column.

In this experiment, a total of 13 nano-SiO_2_ concrete filled GFRP tube column specimens were designed in line with different parameters, including concrete grade, nano-SiO_2_ content, and superplasticizer content, among which the concrete grades include C10, C20, C30, and C40, and the nano-SiO_2_ contents are 0%, 1%, and 3%, respectively. The content of the superplasticizer is 0.8%. The tested GFRP tube was produced by pultrusion process and made of E glass fiber and polyester resin, with a wall thickness (T) of 6 mm, a column height (H) of 250 mm, and a square cross section with side length (L) of 100 mm, as shown in Figure 2. The specimen number and specific parameters are shown in Table 1. Taking GC20-WRA1-NS1 as an example, G represents the GFRP tube, C20 represents a concrete grade of C20, WAR1 represents the superplasticizer included, and NS1 represents 1% of nano-SiO_2_ content. A pair of piezoceramic sensors were made into smart aggregate and embedded in the concrete of GFRP tube, of which one sensor, SS1, acted as an exciter to generate stress wave signals, and the other sensor, SS2, acted as a receiver to receive stress wave signals. The piezoceramic transducers are manufactured by Hengke Technology Co., Ltd., Qinhuangdao City, Hebei Province, China.

### 3.2. Material Properties

**Figure 3:**
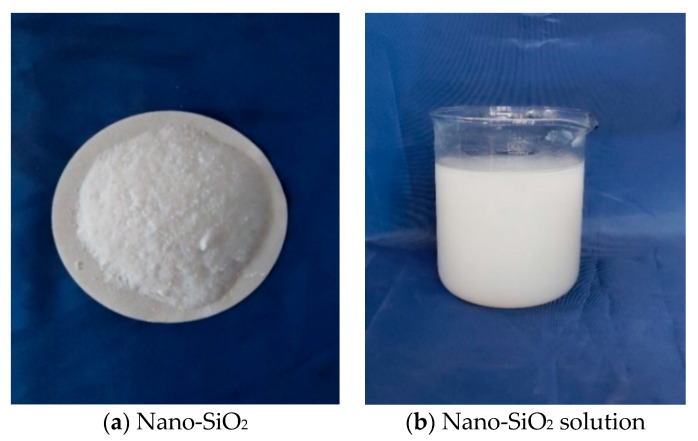
Nano-SiO_2_ powder and solution.

**Figure 4:**
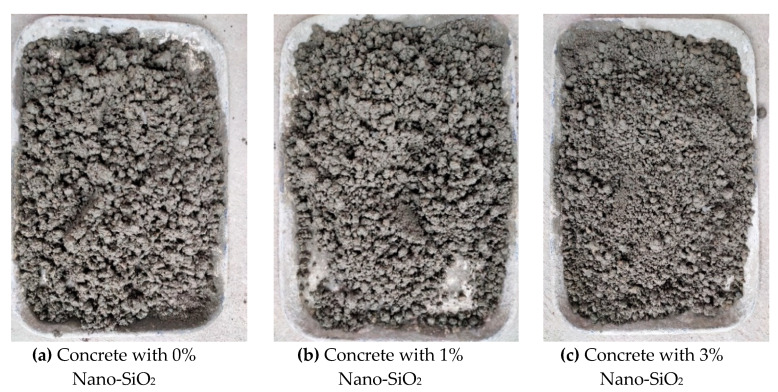
Concrete with different nano-SiO_2_ contents.

**Figure 5:**
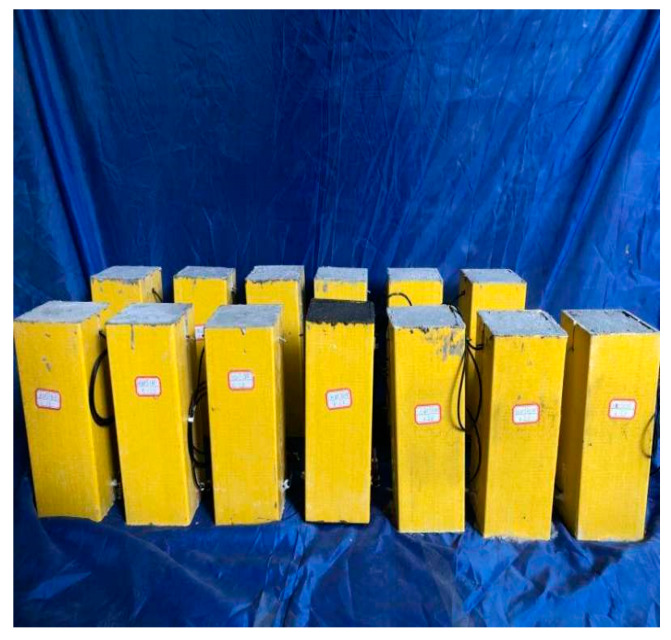
Short GFRP tube concrete column with nano-SiO_2_.

In accordance with GB/T1447-2005 “Test Method for Tensile Properties of Fiber Reinforced Plastics” [69] and GB/T1448-2005 “Test Method for Compressive Properties of Fiber Reinforced Plastics” [70], GFRP material properties were tested by taking standard specimens on the surface of GFRP tube, of which the average compressive strength was 118 MPa, the tensile strength was 550 MPa, and the specific gravity was 2000 kg/m^3^. The mechanical property test of concrete was sampled according to GB/T50080-2002 “Standard for Test Methods of Performance of Common Concrete Mixtures” [71] and configured in the school laboratory. The design codes were C10, C20, C30 and C40, respectively, and the standard cube blocks of 150 mm × 150 mm × 150 mm were cast with the configured concrete. The average compressive strengths of C10, C20, C30 and C40 measured on the testing machine were 17.3, 24.6, 32.5 and 41.3 MPa, respectively. The nano-SiO_2_ adopted hydrophilic gas phase nano-silica Hydrophilic-380, with a specific surface area of 380 mm2/g and a particle size of 7–40 nm. The nano-SiO_2_ powder and the prepared nano-SiO_2_ solution are shown in Figure 3. The concrete added with different nano-SiO_2_ is shown in Figure 4. As can be seen from Figure 4, the concrete mixed with nano-SiO_2_ becomes viscous with a poor fluidity. The superplasticizer was appropriately added so as to reduce the influence of poor fluidity. All experimental specimens are shown in Figure 5.

### 3.3. Loading and Testing Scheme Design

**Figure 6:**
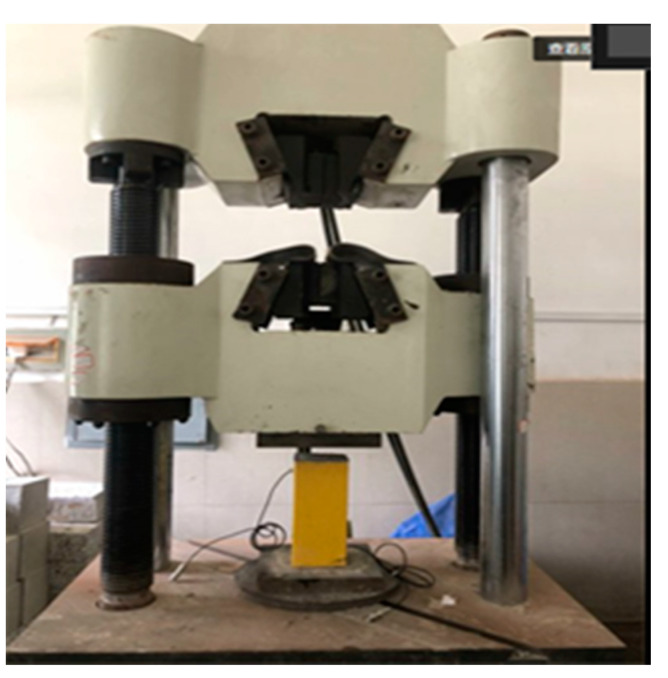
Loading device.

**Figure 7:**
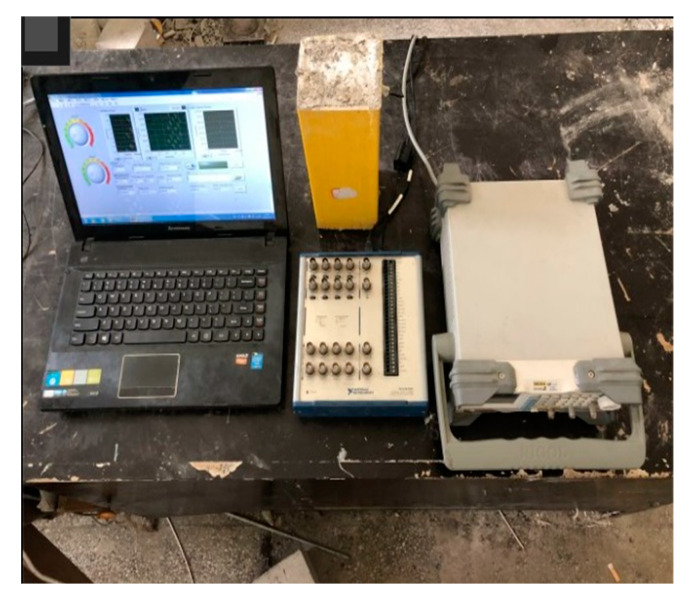
Picture of monitoring system.

The cross-sections of the specimens were polished smooth prior to each test to prevent bias during loading. The 1000 kN electro-hydraulic servo universal testing machine was used for loading, and the displacement control loading mode was adopted, with a speed of 0.5 mm/min. The loading device is shown in Figure 6. The whole health monitoring system consisted of a signal generator, a signal acquisition and analysis system, and a computer, as shown in Figure 7. The signal generator, as the signal source, stimulated SS1 to generate stress waves. The maximum response frequency of the piezoelectric ceramic sensor reached 450 kHz. The signal acquisition and analysis system used a USB-6361 signal collector from the American NI company in order to collect the stress waves received by SS2, and finally stored data in the computer. The frequency sweep mode was put into use in the experiment, with a sweep signal amplitude of 10 V, a frequency range of 100 Hz–50 kHz, and a cycle of 1s. The signal collector obtained the stress waves transmitted in the concrete in real time, which could make the amount of data collected too large. Consequently, according to the loading feedback of the testing machine, the loading was recorded and the stress waves were collected every 30 s until all specimens were completely destroyed. The data collected from all specimens are shown in Table 2.

## 4. Experiment Results and Analysis

### 4.1. Failure Modes

**Figure 8:**
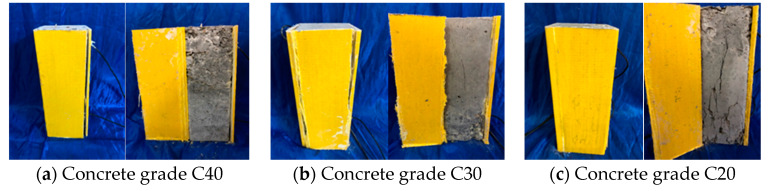
Failure modes of specimens at different concrete grades.

**Figure 9:**
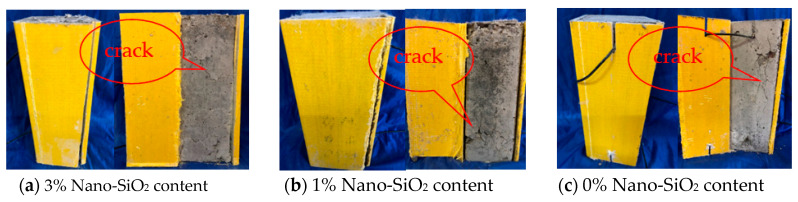
Failure modes of specimens with different nano-SiO_2_ contents.

At the initial stage of loading, there was no obvious change in the GFRP tube wall. With the continuous increase in the load, the top end corner of the specimen was torn, accompanied by the hissing sound of glass fiber tearing; when approaching the ultimate load, the longitudinal tearing became more and more obvious until complete tearing. In order to further observe the damage of the concrete inside the GFRP tube, the torn side of the GFRP tube was stripped. Figure 8 shows the failure modes of specimens at different concrete grades. Figure 9 shows the failure modes of GFRP tube specimens with different nano-SiO_2_ contents.

It can be seen from Figure 8 and Figure 9 that the concrete columns inside the specimens had various degrees of damage, and longitudinal cracks appeared. The C40 concrete column had the smallest crack width and the smallest number of cracks, while the C20 concrete column had the largest crack width and the largest number of cracks; the concrete with 3% nano-SiO_2_ had the smallest crack width and the smallest number of cracks, whereas the concrete without nano-SiO_2_ had the largest crack width and the largest number of cracks.

### 4.2. Load–Displacement Curves

**Figure 10:**
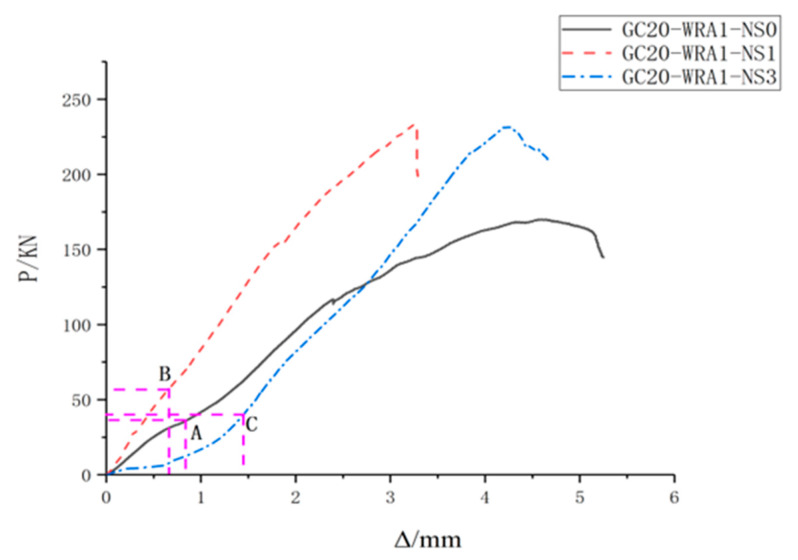
Load–displacement curves of specimens with different nano-SiO_2_ contents.

**Figure 11:**
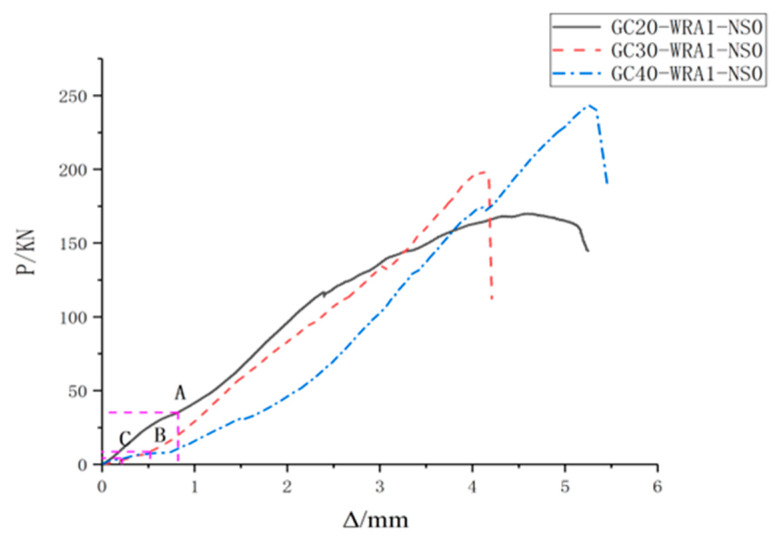
Curves of specimens at different concrete grades.

The experimental data were processed to obtain load–displacement curves, where the horizontal axis (Δ) represents displacement, the vertical axis (P) represents load, and A, B and C represent corresponding loads when the sensor receives the maximum energy. Figure 10 shows the load–displacement curves of specimens with different nano-SiO_2_ contents, and Figure 11 shows the load–displacement curves of specimens at different concrete grades.

As can be seen from Figure 10, when the load is 24.6% of the ultimate load, specimens with 1% nano-SiO_2_ content are damaged; however, when specimens with 0% and 3% nano-SiO_2_ contents are damaged, the loads are respectively 18.7% and 17.9% of the ultimate load. As can be seen from Figure 11, when the applied loads are 3%, 3.2% and 6.5% of the ultimate load, specimens at the concrete grades of C20, C30, and C40 are damaged, respectively.

### 4.3. Load–Energy Index Curves

#### 4.3.1. Load–Energy Index Curves of Specimens with Superplasticizer

**Figure 12:**
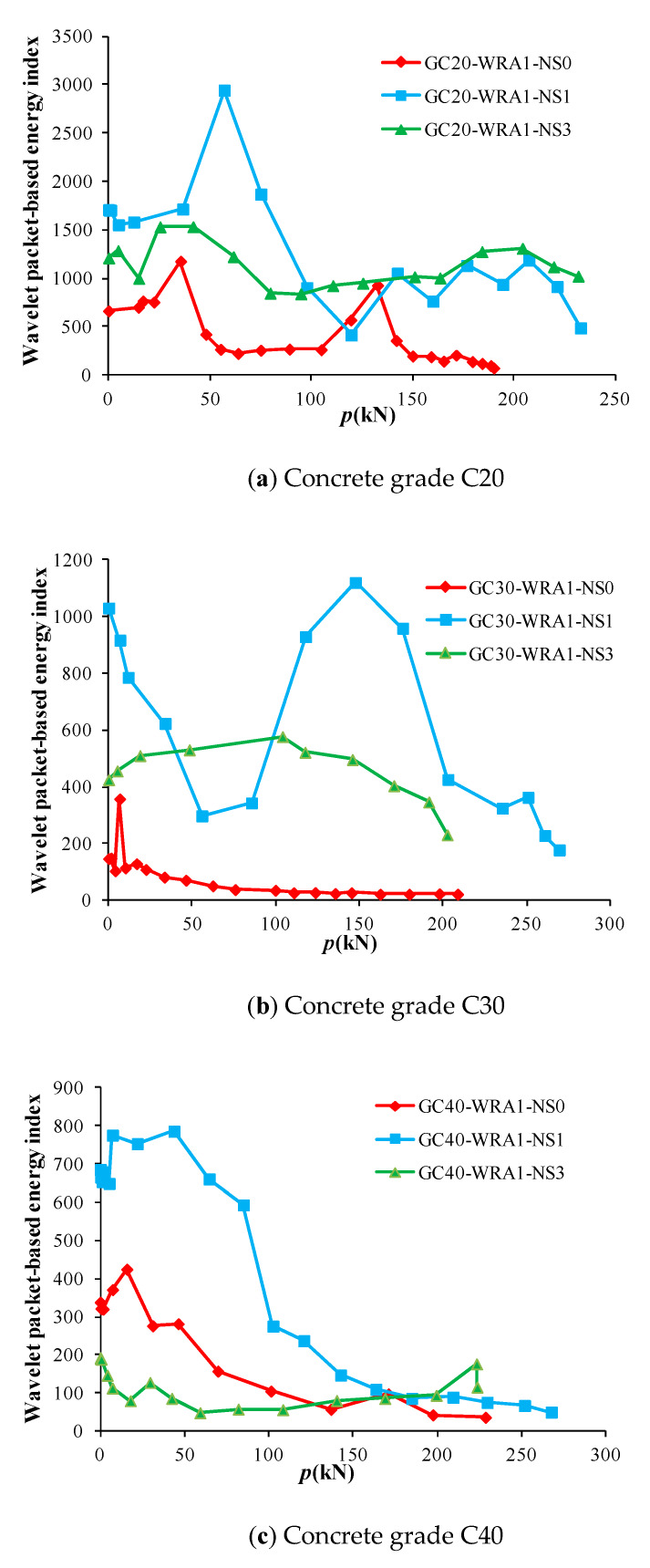
Load–energy index curves of superplasticizer-added specimens with different nano-SiO_2_ contents.

**Figure 13:**
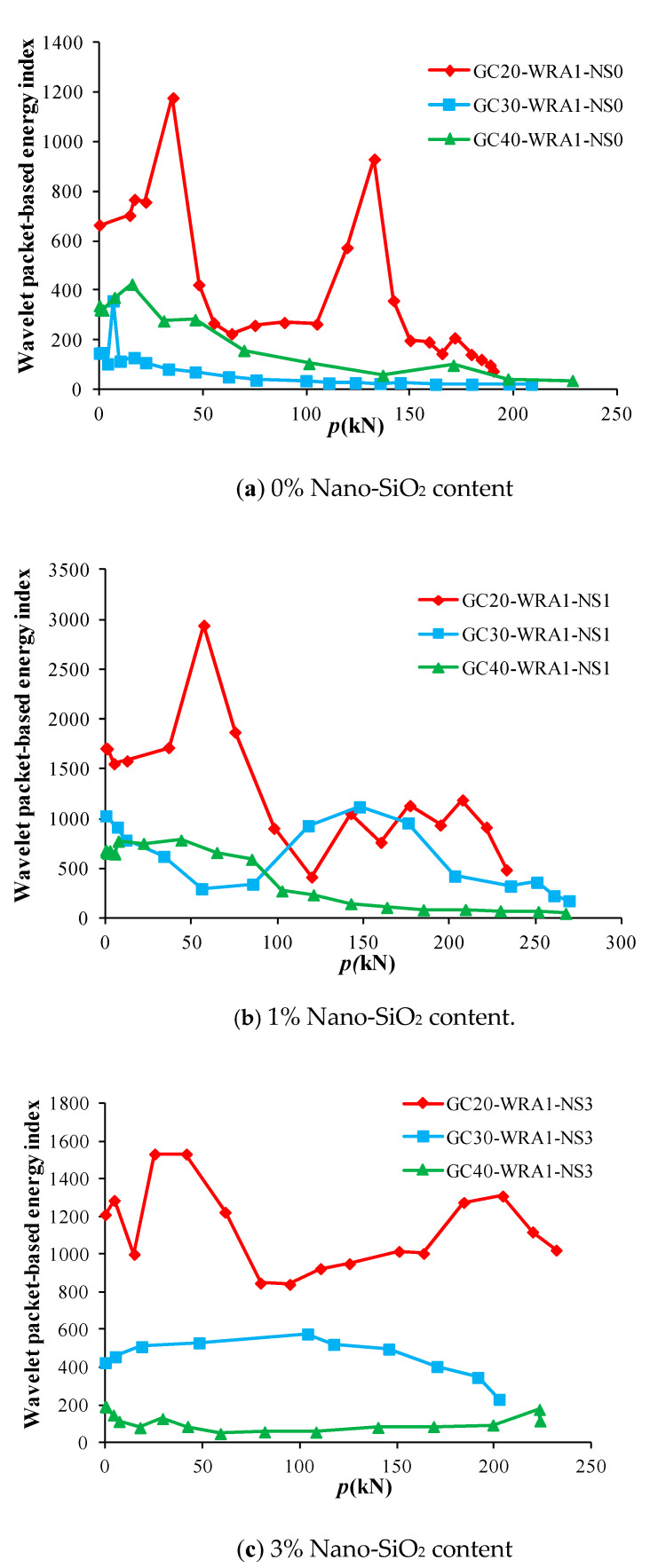
Load–energy index curves of superplasticizer-added specimens at different concrete grades.

The collected data were analyzed and calculated by wavelet packet to obtain the load–energy index curves of specimens with superplasticizer. Figure 12 shows the load–energy index curves of superplasticizer-added specimens with different nano-SiO_2_ contents, and Figure 13 shows the load–energy index curves of superplasticizer-added specimens at different concrete grades.

At the initial stage of loading, the compression and compaction of concrete in GFRP tube played a major role, and the energy of the received signal increased and gradually reached the maximum. Then, with the further increase in load, the concrete was damaged under compression, and micro-cracks began to develop, resulting in a gradual decrease in energy. As can be seen from Figure 12, specimens with 1% nano-SiO_2_ content have the highest initial energy, and can bear the largest load with the increase in load until being destroyed. However, when the concrete inside specimens is damaged, specimens with 1% nano-SiO_2_ content have the maximum energy attenuation slope value of 4.71, whereas specimens with 3% nano-SiO_2_ content have the minimum energy attenuation slope value of 0.53., implying that adding 3% nano-SiO_2_ content to concrete can effectively delay the development of damage.

As it can be seen from Figure 13, during the entire loading period, the energy of superplasticizer-added specimens at a concrete grade of C20 is basically higher than that at the concrete grades of C30 and C40, indicating that there are relatively few cracks in C20 concrete. Nevertheless, in the process of increasing load, the energy attenuation slope value of specimens at a concrete grade of C20 is the largest, which is 6.31. However, the energy attenuation slope value of specimens at the concrete grades of C30 and C40 are relatively smaller. This indicates that after the addition of superplasticizer, the higher the concrete grade of the specimen, the slower the development of cracks in concrete, and the stronger the resistance to damage.

#### 4.3.2. Load–Energy Index Curves of Specimens without Superplasticizer

**Figure 14:**
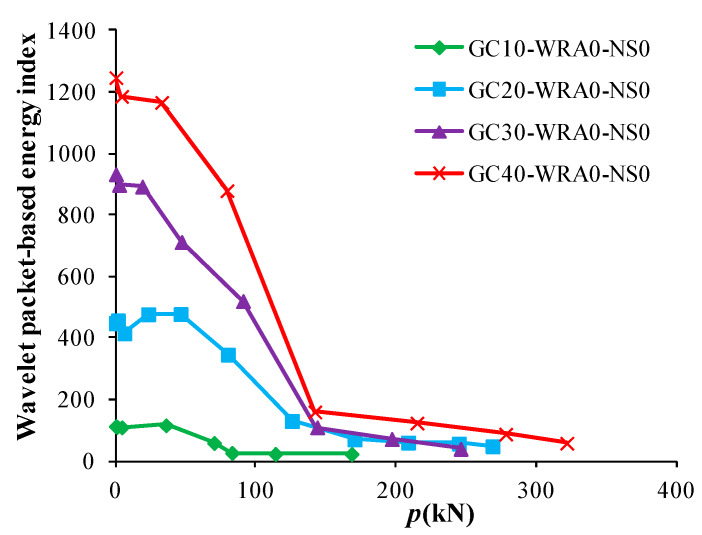
Load–energy index curves of specimens without superplasticizer at different concrete grades.

Figure 14 shows the load–energy index curves of specimens without superplasticizer at different concrete grades. As it can be seen from Figure 14, when no superplasticizer is added, the initial energy of specimen at a concrete grade of C10 is the smallest, whereas that at a concrete grade of C40 is the largest. With the increase in load, the energy attenuation of specimens at a concrete grade of C40 is the fastest, indicating that when superplasticizer is not added, the higher the concrete grade, and the faster the development of specimen damage.

### 4.4. Load–Damage Index Curves

**Figure 15:**
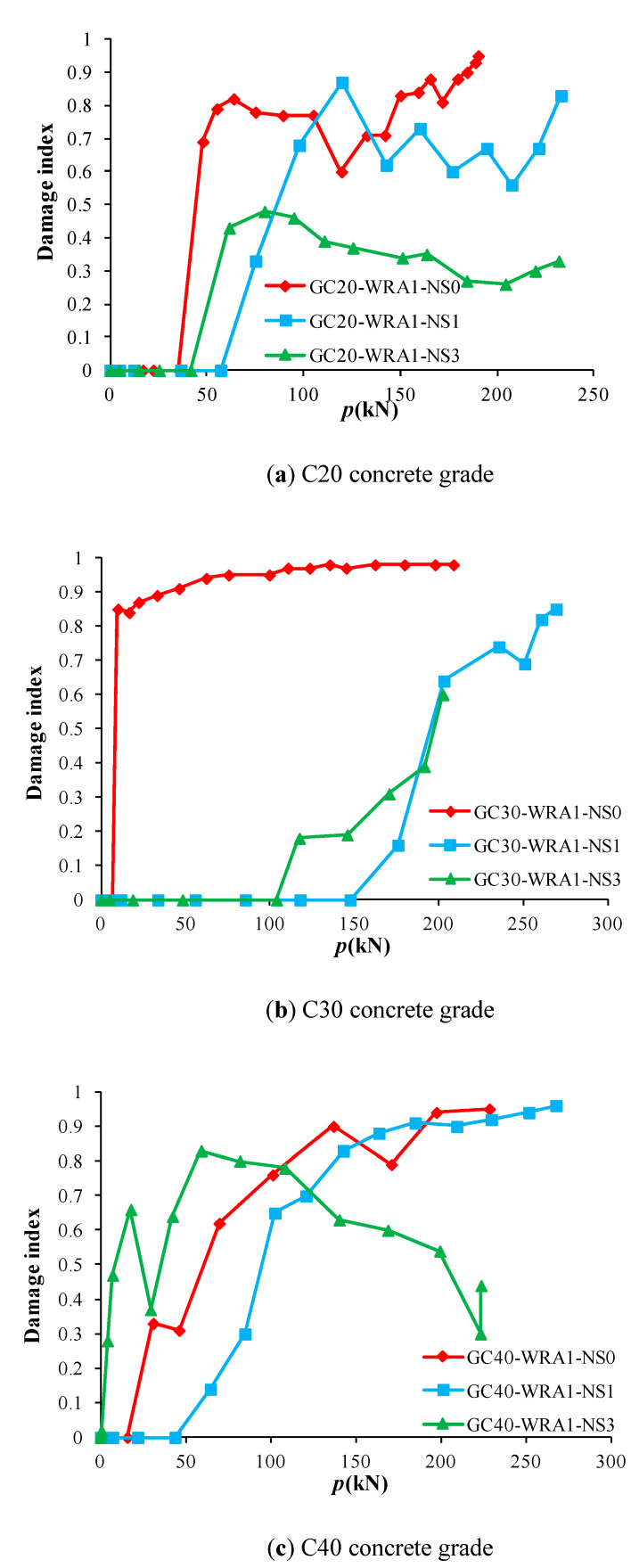
Index curves of specimens with different nano-SiO_2_ contents.

**Figure 16:**
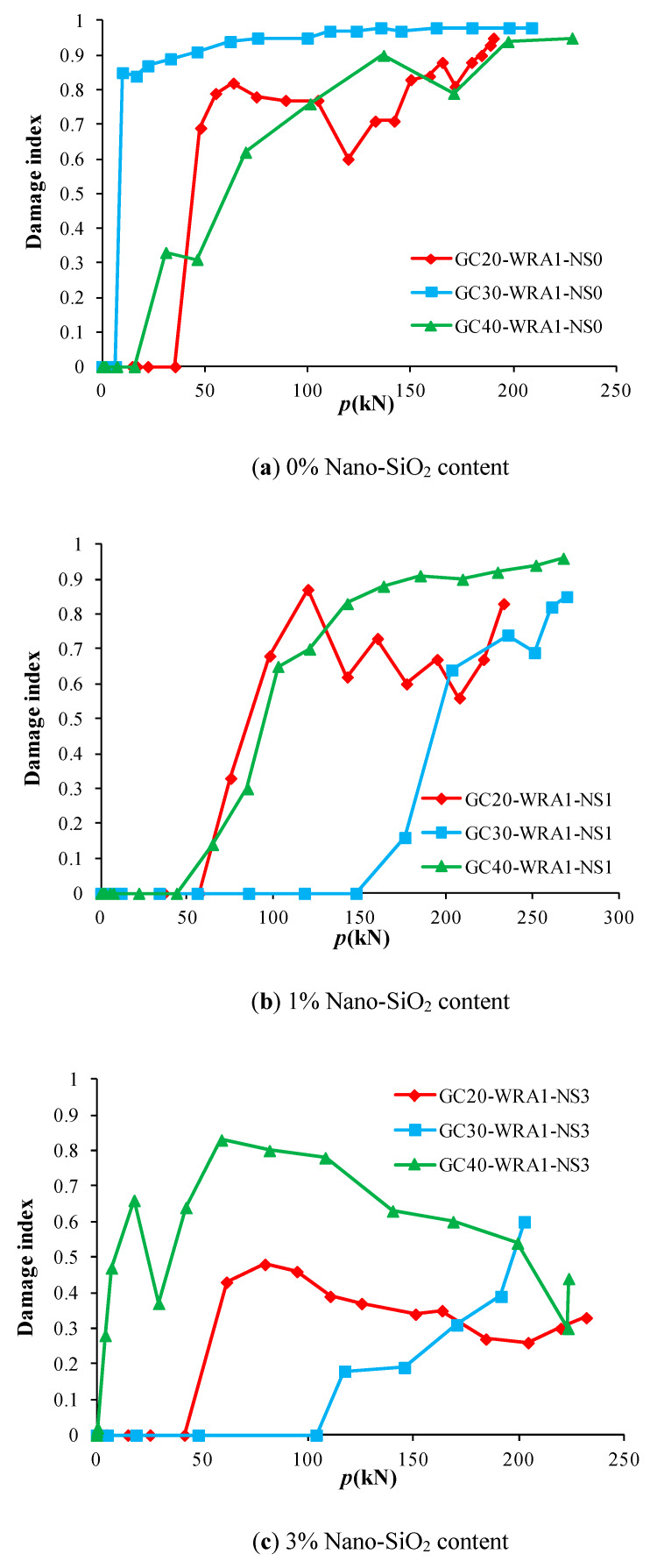
Load–damage index curves of specimens at different concrete grades.

The load–damage index curve of each specimen can be gained from Table 2. Figure 15 shows the load–damage index curves of specimens with different nano-SiO_2_ contents, and Figure 16 shows the load–damage index curves of specimens at different concrete grades.

As can be seen from Figure 15, specimens with 1% nano-SiO_2_ content have the highest load at the beginning of damage, indicating that the damage of specimens with 1% nano-SiO_2_ content occurs the latest, followed by specimens with 3% nano-SiO_2_ content, yet the damage without the nano-SiO_2_ specimen occurs the fastest. With the increase in load, the damage index of specimens without nano-SiO_2_ increases rapidly to over 0.8, followed by specimens with 1% nano-SiO_2_ content, while the damage index of specimens with 3% nano-SiO_2_ content changes the slowest, demonstrating that the damage of specimens without nano-SiO_2_ content develops the fastest. The damage index of each specimen changes relatively slowly when reaching 0.6. As can be seen from Figure 16, the change rate of the damage index of specimens at a concrete grade of C20 is the largest, while that at a concrete grade of C40 is the smallest, implying that the higher the concrete grade, the lower the damage rate.

## 5. Conclusions

In this paper, a new method of damage identification using piezoelectric transducers to monitor nano-SiO_2_ concrete filled glass fiber reinforced polymer (GFRP) tube column is mentioned. The results show that the damage index based on wavelet packet energy can effectively monitor the damage of concrete in GFRP tube, and the damage identification method based on wave analysis is feasible and effective. Based on the analysis of the experiment results, the conclusions can be summarized as follows:

1. The stress wave method using piezoceramic transducers can monitor the damage of the concrete in the glass fiber reinforced polymer (GFRP) tube well. The failure modes of specimens and the development trend of load–displacement curves are well reflected in the changes in energy and damage indices.
2. At the initial stage of load, when the concrete is under compression and compaction, the stress wave energy increases; however, as the load increases, cracks appear in the concrete and develop, the energy decreases, and the damage index increases from 0 to 1.
3. Specimens with 1% nano-SiO_2_ content have the largest initial energy and the maximum ultimate load. When the concrete inside specimens is damaged, the energy change rate of specimens with 3% nano-SiO2 content is the smallest, indicating that adding 3% nano-SiO_2_ content to concrete can effectively delay the development of damage.
4. After the addition of superplasticizer, the higher the concrete grade of the specimen, the slower the development of cracks in concrete, and the stronger the resistance to damage. When no superplasticizer is added, the higher the concrete grade is, the faster the damage of the specimen develops.
5. The damage index of specimens with 0% nano-SiO_2_ content develops the fastest. After the damage index reaches 0.6, the damage index of each specimen changes relatively slowly.

The structure health monitoring technology using piezoelectric transducers can monitor the health status of the structure in real time and produce early warnings of potential risks, which is suitable for the long-term monitoring of high-rise buildings and large-scale infrastructure. In future research, the interface peeling of GFRP tubular concrete columns using piezoelectric tranducers and the monitoring and identification of internal concrete defects will be studied. Different characteristic parameters of signals, such as signal amplitude, signal energy and signal power spectral density, will be selected as research objects in the time and frequency domain, and various damage indexes based on characteristic parameters will be proposed.

## Figures and Tables

**Table 1:** Specimen number and specific parameters.

Specimen Name	Height H (mm)	Length L (mm)	Thickness T (mm)	Concrete Grade C (MPa)	Nano SiO_2_ Content	Polycarboxylate Superplasticizer
GC20-WRA1-NS0	250	100	6	C20	0	0.8%
GC20-WRA1-NS1	250	100	6	C20	1%	0.8%
GC20-WRA1-NS3	250	100	6	C20	3%	0.8%
GC30-WRA1-NS0	250	100	6	C30	0	0.8%
GC30-WRA1-NS1	250	100	6	C30	1%	0.8%
GC30-WRA1-NS3	250	100	6	C30	3%	0.8%
GC40-WRA1-NS0	250	100	6	C40	0	0.8%
GC40-WRA1-NS1	250	100	6	C40	1%	0.8%
GC40-WRA1-NS3	250	100	6	C40	3%	0.8%
GC10-WRA0-NS0	250	100	6	C10	0	0
GC20-WRA0-NS0	250	100	6	C20	0	0
GC30-WRA0-NS0	250	100	6	C30	0	0
GC40-WRA0-NS0	250	100	6	C40	0	0

**Table 2:** Specimen loading and data collection.

**GC20-WRA1-NS0**	**Load(KN)**	**0**	**14.6**	**16.9**	**22.3**	**35.5**	**47.9**	**55.2**	**63.8**	**75.0**	**89.2**	**104.9**	**119.5**	**132.7**	**141.9**	**150.0**	**159.1**	**165.3**	**171.5**	**179.5**	**184.3**	**188.6**	**190.1**
	**Wavelet packet-based energy index**	664.2	702.9	767.0	756.4	1176.7	423.0	268.0	225.1	259.0	270.4	264.7	572.6	929.3	359.2	198.4	191.9	145.5	208.4	141.7	121.6	99.1	75.1
	**Damage index**	0	0	0	0	0	0.69	0.79	0.82	0.78	0.77	0.77	0.60	0.71	0.71	0.83	0.84	0.88	0.81	0.88	0.90	0.93	0.95
**GC20-WRA1-NS1**	**Load(KN)**	0	0.3	0.9	4.8	12.3	36.5	57.0	75.1	97.7	119.7	142.5	160.0	176.8	194.5	207.4	221.3	232.9					
	**Wavelet packet-based energy index**	1709.4	1705.3	1705.3	1553.6	1582.2	1716.5	2943.8	1870.7	905.3	415.9	1053.0	764.4	1133.7	937.1	1190.5	916.4	488.4					
	**Damage index**	0	0	0	0	0	0	0	0.33	0.68	0.87	0.62	0.73	0.60	0.67	0.56	0.67	0.83					
**GC20-WRA1-NS3**	**Load(KN)**	0	4.6	14.8	25.3	41.6	61.5	79.7	94.8	110.6	125.4	150.9	163.5	184.2	204.2	219.6	231.7						
	**Wavelet packet-based energy index**	1211.0	1286.3	1000.5	1532.6	1533.0	1224.0	847.8	840.6	924.3	950.4	1015.9	1005.0	1275.8	1309.9	1118.9	1022.5						
	**Damage index**	0	0	0	0	0	0.43	0.48	0.46	0.39	0.37	0.34	0.35	0.27	0.26	0.30	0.33						
**GC30-WRA1-NS0**	**Load(KN)**	0	1.4	3.9	6.6	10.0	16.7	22.3	33.3	46.2	62.3	75.6	99.7	110.7	123.5	135.4	145.2	162.3	179.7	197.7	208.6		
	**Wavelet packet-based energy index**	147.3	149.2	104.3	358.2	114.8	129.7	109.1	82.2	71.4	51.3	38.3	35.8	27.0	27.7	24.8	27.4	22.0	22.1	23.4	22.2		
	**Damage index**	0	0	0	0	0.85	0.84	0.87	0.89	0.91	0.94	0.95	0.95	0.97	0.97	0.98	0.97	0.98	0.98	0.98	0.98		
**GC30-WRA1-NS1**	**Load(KN)**	0	6.7	11.7	33.7	55.7	85.4	117.7	147.5	175.8	202.9	235.4	250.7	260.7	269.4								
	**Wavelet packet-based energy index**	1031.1	917.9	786.3	623.9	298.2	343.9	930.3	1121.1	959.9	426.3	325.7	363.7	228.9	178.3								
	**Damage index**	0	0	0	0	0	0	0	0	0.16	0.64	0.74	0.69	0.82	0.85								
**GC30-WRA1-NS3**	**Load(KN)**	0	5.1	18.7	48.2	103.9	117.4	145.7	170.5	191.4	202.4												
	**Wavelet packet-based energy index**	425.7	456.5	510.5	530.7	577.4	522.2	496.8	404.3	346.8	231.1												
	**Damage index**	0	0	0	0	0	0.18	0.19	0.31	0.39	0.60												
**GC40-WRA1-NS0**	**Load(KN)**	0	0.4	0.5	0.6	1.7	7.2	15.7	31.0	46.2	69.7	101.1	136.7	170.8	197.2	228.3							
	**Wavelet packet-based energy index**	338.7	322.5	322.3	321.6	320.9	371.9	425.3	276.9	282.3	158.0	105.5	57.8	98.3	43.0	36.7							
	**Damage index**	0	0	0	0	0	0	0	0.33	0.31	0.62	0.76	0.90	0.79	0.94	0.95							
**GC40-WRA1-NS1**	**Load(KN)**	0	0.1	0.2	0.3	1.2	2.3	5.2	7.2	21.9	43.7	64.5	84.8	102.4	120.7	142.5	163.4	184.7	209.1	229.4	251.5	267.4	
	**Wavelet packet-based energy index**	686.3	684.8	670.3	666.1	653.9	679.7	649.5	776.5	754.0	786.7	661.7	594.1	276.0	237.5	147.7	110.2	86.4	89.6	76.0	67.7	50.4	
	**Damage index**	0	0	0	0	0	0	0	0	0	0	0.14	0.30	0.65	0.70	0.83	0.88	0.91	0.90	0.92	0.94	0.96	
**GC40-WRA1-NS3**	**Load(KN)**	0	0.1	0.5	4.1	7.1	17.8	29.4	42.3	59.1	81.8	108.2	140.1	168.7	199.2	223.1	223.4						
	**Wavelet packet-based energy index**	192.8	190.5	189.2	147.0	113.4	80.1	128.0	86.4	48.4	58.1	56.6	80.7	85.9	94.2	176.8	115.8						
	**Damage index**	0	0.01	0.02	0.28	0.47	0.66	0.37	0.64	0.83	0.80	0.78	0.63	0.60	0.54	0.30	0.44						
**GC10-WRA0-NS0**	**Load(KN)**	0	0.1	0.1	0.8	4.2	35.7	70.0	82.8	113.7	168.1												
	**Wavelet packet-based energy index**	113.3	113.7	113.5	111.4	109.7	116.3	58.9	26.5	23.2	22.9												
	**Damage index**	0	0	0.06	0.08	0.10	0.08	0.59	0.92	0.96	0.96												
**GC20-WRA0-NS** **0**	**Load(KN)**	0	0.1	1.4	6.1	23.0	46.2	79.9	125.5	170.2	208.4	244.6	268.7										
	**Wavelet packet-based energy index**	449.2	447.5	457.3	415.9	477.8	478.1	345.9	130.6	70.5	60.7	57.3	48.0										
	**Damage index**	0	0	0	0	0	0	0.28	0.34	0.47	0.75	0.90	0.93										
**GC30-WRA0-NS0**	**Load(KN)**	0	2.3	18.8	46.9	90.7	143.6	196.8	245.9														
	**Wavelet packet-based energy index**	933.1	898.5	893.2	712.6	520.6	110.1	72.0	40.0														
	**Damage index**	0	0.03	0.25	0.32	0.44	0.45	0.61	0.65														
**GC40-WRA0-NS0**	**Load(KN)**	0	4.1	32.7	79.2	141.9	214.8	278.0	321.6														
	**Wavelet packet-based energy index**	1247.3	1186.1	1166.8	879.8	160.0	123.6	88.0	58.4														
	**Damage index**	0	0.05	0.26	0.34	0.87	0.90	0.94	0.97														
